# Effects of Developmental Care on Neurodevelopment of Preterm Infants

**Published:** 2020

**Authors:** Farin Soleimani, Nadia Azari, Hesam Ghiasvand, Shiva Fatollahierad

**Affiliations:** 1 1.Pediatric Neurorehabilitation Research Center, University of Social Welfare and Rehabilitation Sciences, Tehran, Iran; 2 Health Economics Group, Medical School, Saint Luke's Campus, University of Exeter, Exeter, UK.; 3 3.Faculty Member, Social Determinants of Health Research Center, University of Social Welfare and Rehabilitation Sciences, Tehran, Iran

**Keywords:** Preterm infants, Developmental care, NICU, Interventions, Bayley Scales

## Abstract

**Objectives:**

The aim of this study protocol is to systematically review the literature to examine the effects of developmental care on preterm infants’ neurodevelopment in the neonatal intensive care unit (NICU).

**Materials & Methods:**

Studies will be retrieved through searching the following databases: Web of Science, PubMed, EMBASE (Ovid), Cochrane Central Register of Controlled Trials (CENTRAL), CINAHL (EBSCO), and Scopus.

Randomized controlled trials will be included with randomization at either individual or cluster level. The primary outcome will be to evaluate the effect of developmental care on the mental and motor development of NICU neonates. The secondary outcome will be neonatal weight gain and length of stay during NICU hospitalization. The assessment tool for the development should be the Bayley Scales of Infant and Toddler Development, in any of the first, second and third editions.

Preferred Reporting Items for Systematic Reviews and Meta-Analyses (PRISMA) will be employed to identify relevant articles and report the screening process. The agreement between two experts in developmental neonatology will be reached in selecting all studies. Afterward, data will be extracted and compared by two reviewers. Any discrepancies in the extracted data will be discussed to reach a consensus. The extracted data will be imported to Review Manager 5.3 by one reviewer. Finally, the risk of bias for all selected studies will be independently evaluated by two reviewers using the Cochrane Collaboration’s tool.

A meta-analysis will be performed to assess the possible quantitative impact of developmental interventions on the desired primary and secondary objectives. A random effect will be used if the I-square statistics is equal or more than 75%; otherwise, a fixed effect will be applied. Publication bias will be assessed using Egger’s test and illustration with the funnel plot. The Standardized Mean Difference (SMD) with 95% confidence interval will be estimated through Metan command in STATA 14. The method provided in the Cochrane handbook will be used in this statistical analysis. The significance level will be 0.05.

## Introduction

The birth rate of preterm infants has increased worldwide over the last few decades with technological advances. About 15 million preterm infants are born every year in the world; in other words, one in 10 infants is born prematurely (1). Moreover, the use of treatments such as antenatal steroid therapy, surfactant, positive pressure ventilation, and neonatal intensive care has increased the survival rate of preterm infants. Prematurity and its complications are the main cause of death during infancy and also the second cause of death among children under five years of age (2), with nearly one million deaths in 2015. 

Prematurity is also the leading cause of long-term developmental disabilities (3). Prematurity can cause a range of long-term and possibly lifelong complications in the metabolic, respiratory and nervous systems of individuals, as well as in their physical health. Complications related to prematurity are responsible for a part of the burden of chronic diseases in adults (4). Thus, focus on care for preterm infants hospitalized in neonatal intensive care units (NICU) has shifted from increased survival rates to reduced complications and improved quality of life (5-7). In the third trimester of pregnancy, important structures like the thalamus, the cortex, and the cerebellum are produced and the neuronal synaptic network is maximally developed. Therefore, from 24 to 40 weeks of gestation, the brain experiences a critical period of growth (8, 9). Studies have shown that exogenous and endogenous experiences of preterm infants in NICU can lead to developmental disorders (10, 11). In fact, NICU has harmful sensory effects on the immature brain of preterm infants and impairs their future development. Most infants admitted to NICU experience consequences such as separation from parents, recurrent aggressive and painful procedures, and excessive sensory stimulation, which are in contrast to expectations of the nervous system and lead to considerable changes in this system (12). Inappropriate sleep patterns in NICU, inattention and no proper response to the newborn’s behaviors, positional changes from full intrauterine flexion to supine and extension, rapid and inappropriate handling, invasive methods, excessive light and sound in the environment, and lack of enhancement of sucking skills have adverse effects on the immature nervous system and cause disruption in the infant’s development (13, 14). 

Early intervention has been used more in recent years, aiming at improving developmental outcomes in preterm infants. For early intervention, various methods have been proposed and implemented by specialists over the past few decades. Among such methods, developmental care (DC), kangaroo mother care (KMC), infant massage, and Neonatal Individualized Developmental Care and Assessment Program (NIDCAP) are mostly noticed. Complex biological, medical and environmental factors interfering with the newborn’s development have led to the formation of interventions with different components and services offered by various disciplines (15). One program may have more impact on long-term complications and focus more on neurodevelopmental care and stress reduction in the NICU environment as well as on individual and behavioral care programs such as NIDCAP designed to improve development of preterm infants. However, some interventions may focus on communicating with parents of preterm infants to provide psychological support for parents and practical guidance on caring for their infants (16).

The effectiveness of developmental care in preterm infants has been studied in several systematic reviews (16-23). In the studies by Symington and Pinelli (19, 20) and Lavallée (21, 22), the effectiveness of five developmental care interventions in NICU was addressed. In other systematic reviews, post-NICU discharge interventions were studied. Nevertheless, developmental outcomes resulting from these interventions were not measured by a single instrument with high sensitivity and specificity (19, 20, 22). This has gained growing interest as several clinical trials have been conducted in the field during recent years. However, results of trials have been mostly different and sometimes contradictory, and no definite conclusion has been obtained on the effectiveness of developmental care in NICU. Therefore, there is a need for conducting a systematic review in this regard.

This study aims to review the literature to determine the effects of developmental care in NICU on neurodevelopmental outcomes of preterm infants assessed using the Bayley Scales of Infant and Toddler Development. 

## Methods &analyses

To perform this review protocol, the Preferred Reporting Items for Systematic Reviews and Meta-Analyses for Protocols 2015 (PRISMA-P 2015) checklist will be used (24) and has been registered in the International Prospective Register of Systematic Reviews with the registration number CRD42018085386.

**Eligibility criteria: **The following eligibility criteria will be encountered in selected studies in this review protocol: study design (including publication, language, and year), participants, interventions, comparators, and outcomes.

**Study design**: Randomized controlled trials with randomization will be included at either individual or cluster level to evaluate the effectiveness of developmental care in NICU on developmental outcomes in preterm infants.

Observational studies such as quasi-experimental (no randomization or no comparative group) and Cohort studies in addition to descriptive and case-control studies will be excluded. The research design provides the highest level of evidence and thus only randomized controlled trials will be included in the review protocol. There will be no time and language restrictions in the search strategy; however, published studies with English abstracts will be only included. 

**Participants**: Studies on preterm infants (≤37 weeks of gestation) will be included in the review. However, infants with intra-ventricular hemorrhage greater than grade II, brain malformation or any other health condition influencing their development will be excluded. 

**Interventions**: Depending on the desired outcomes, early intervention may focus on different aspects such as cognitive, language and motor development. Developmental care with an emphasis on the environment and the infant is designed to minimize stress for the infant in NICU. It may include sensory stimulation, parental participation, and modification of the environment that may have a large effect on long-standing morbidity. In this study, developmental care is defined as care in NICU designed to improve either cognitive or motor development, or both; these interventions can be carried out by caregivers in NICU, parents, or both.

Studies with post NICU components will be excluded the review. Moreover, interventions designed to reduce pain or improve nutrition and parents’ psychological support will be excluded from the review. Authors of selected studies will be contacted if required to obtain details about interventions.

**Comparator**: A non-exposed control group or a group exposed to different interventions will be included in the systematic review.

**Outcome**: Our primary outcome will be the long term effect of developmental care in NICU neonates on their cognitive and motor development. The secondary outcome will be short term outcome such as weight on discharge and hospital stay length in NICU. 

The main attention point is selection of one age-appropriate standardized scale for better comparison, thus the assessment tool of long term outcome should be the Bayley Scales of Infant and Toddler Development (BSID) in any of the first, second or third edition(25-27). The Bayley Scales of Infant Development is the gold standard for assessment of developmental delay and disorder with high validity and reliability of children from 1 to 42 months of age providing separate scores for cognitive, language and motor function. The Bayley has been reformed in 1993 (BSID II) and 2006 (BSID III)(26, 27). 

**Information sources: **A list of keywords to be used individually or in combination will be created by two experts on developmental neonatology to conduct the search. Studies will be retrieved through searching the following databases: PubMed, MEDLINE (Ovid), EMBASE (Ovid), Cochrane Central Register of Controlled Trials (CENTRAL), CINAHL (EBSCO), Web of Science and Scopus. Moreover, for identification of trials which are presently ongoing, the website clinicaltrials.gov will be re-checked. In order to recognize other possible relevant studies, references of included studies and previous related review articles will be screened. Text words and medical subject headings (MeSH/thesaurus terms) will be used to search databases. 

**Search strategy**: Our initial search syntax for PubMed is demonstrated in Table 1. 

**Screening of studies: **Duplicates of studies will be first investigated by two separate reviewers and then compared to affirm proper deletion of studies. Titles and abstracts will be screened to select relevant studies. Full texts of potentially eligible articles meeting the inclusion criteria will be read by three reviewers for inclusion in the review. Any disagreement about selecting articles will be resolved through discussion and consensus. Data extraction of included studies will be carried out by the authors using a form designed for the review. Any discrepancies in extracted data will be discussed to reach a consensus. In a report form, the number of included and excluded studies along with explanations for exclusion will be noted. 

**Data extraction: **Data extraction of included studies will be carried out by the authors using a form designed for the review. Any discrepancies in extracted data will be discussed to reach a consensus. Extracted data will be imported to Review Manager 5.3 by one reviewer.

For each study, data will be collected on the following domains: author, date of publication, design of study, location and setting of study, date of intervention, sample size, gestational age, birth weight, co-morbidities, inclusion and exclusion criteria, frequency of intervention delivery, duration of intervention, co-interventions, outcomes, assessment tools of outcomes, results, method of allocation and randomization, blinding of participants and outcome assessors, exclusion of participants after randomization and proportion of losses to follow-up.

**Assessment of risk of bias: **The risk of bias for all included studies will be independently evaluated by two reviewers using the Cochrane Collaboration’s tool for assessing risk of bias in randomized trials (28). Any disagreement will be resolved by discussion. The risk of bias tool assesses the following criteria: random sequence generation (checking for possible selection bias), allocation concealment (checking for possible selection bias), blinding of participants and personnel (checking for possible performance bias), blinding of outcome assessment (checking for possible detection bias), incomplete outcome data (checking for possible attrition bias through withdrawals, dropouts, and protocol deviations), selective reporting (checking for possible reporting bias) and other sources of bias. The reviewers’ judgments will be categorized as ‘low risk’ of bias, ‘high risk’ of bias or ‘unclear risk’ of bias.

**Data synthesis and statistical analysis: **If selected studies are satisfactorily homogeneous for developmental, weight gain and length of hospital stay outcomes, a meta-analysis will be performed. A random effect will be chosen if I-square - as the heterogeneity criteria - is equal to or greater than 75%; otherwise, a fixed effect will be applied. Publication bias will be assessed using Egger’s test and illustration with the funnel plot. The Standardized Mean Difference (SMD) with 95% confidence interval will be estimated through Metan command in STATA 14 (29, 30). 

If there are multi-arm intervention and control groups in a trial, sample size, mean and standard deviation will be calculated for obtaining a single intervention and control group. The method provided by the Cochrane handbook will be used in this statistical analysis (28, 29). 

The significance level will be 0.05. Subgroup analysis will be on type of intervention, gestational age, and birth weight.

**Missing data: **Missing data will be obtained by contacting corresponding authors of articles.

**Table 1:** PubMed search strategy

Search number	Query
#1	"Intensive Care Units, Neonatal"[Mesh] OR "neonatal ICU"[tiab] OR "neonatal intensive care units"[tiab] OR "neonatal intensive care unit"[tiab] OR " newborn intensive care units"[tiab] OR "newborn intensive care unit"[tiab] OR "NICU"[tiab] OR "newborn ICU"[tiab]
#2	"Intensive Care, Neonatal"[Mesh] OR "neonatal intensive care"[tiab] OR "newborn intensive care"[tiab]
#3	"Infant, Premature"[Mesh] OR "Infant, Extremely Premature"[Mesh] OR “Premature Birth"[Mesh] OR "premature"[tiab] OR "preterm"[tiab] OR "neonatal prematurity"[tiab]
#4	"Infant, Very Low Birth Weight"[Mesh] OR "Infant, Low Birth Weight"[Mesh] OR "Infant, Extremely Low Birth Weight"[Mesh] OR "low birth weight"[tiab] OR “VLBW”[tiab] OR “LBW”[tiab]
#5	#1 OR #2 OR #3 OR #4
#6	"bayley"
#7	#5 AND# 6

## Discussion

This study will be the first systematic review that assesses the effect of NICU developmental care on neurodevelopment in preterm infants. According to findings of this systematic review, by abstracting results and presenting conclusions, interventional decisions can be improved. Further, by examining strengths and weaknesses of studies using meta-analysis, effectiveness of interventions will be elaborated and also a relatively straightforward conclusion will be achieved about long-term outcomes of developmental care in NICU for promoting preterm infants’ neurodevelopment during hospitalization.

In the case of small studies, which have relative heterogeneity and limited quality, deficiency in existing evidence becomes clearer and suggestions such as research design, methods, sample size, and issues of test power can be recommended for further research. 

The results of this systematic review, by briefing areas (if there is sufficient evidence in this area), improving health interventions and, lacking sufficient evidence will mostly require the need to focus on next research activities and allocate more resources.

**In conclusion**

The clinical application could be enhanced to improve results and specifically neurodevelopment in preterm infants, with this knowledge.

## References

[1] Liu L, Johnson HL, Cousens S, Perin J, Scott S, Lawn JE (2012). Global, regional, and national causes of child mortality: an updated systematic analysis for 2010 with time trends since 2000.

[2] (2015). WHO recommendations on interventions to improve preterm birth outcomes: evidence based.

[3] Liu L, Oza S, Hogan D, Chu Y, Perin J, Zhu J (2016). Global, regional, and national causes of under-5 mortality in 2000-15: an updated systematic analysis with implications for the Sustainable Development Goals. Lancet..

[4] Blencowe H, Lee ACC, Cousens S, Bahalim A, Narwal R, Zhong N (2013). Preterm birth-associated neurodevelopmental impairment estimates at regional and global levels for 2010. Pediatric research..

[5] Fanaroff AA, Wright LL, Stevenson DK, Shankaran S, Donovan EP, Ehrenkranz RA (1995). Very-low-birth-weight outcomes of the National Institute of Child Health and Human Development Neonatal Research Network, May 1991 through December 1992. American Journal of Obstetrics and Gynecology..

[6] Hack M, Taylor HG, Klein N, Eiben R, Schatschneider C, Mercuri-Minich NJNEJoM (1994). School-age outcomes in children with birth weights under 750 g.

[7] Ornstein M, Ohlsson A, Edmonds J, Asztalos EJAP (1991). Neonatal follow‐up of very low birthweight/extremely low birthweight infants to school age: a critical overview.

[8] Volpe JJJTLN (2009). Brain injury in premature infants: a complex amalgam of destructive and developmental disturbances.

[9] Pickler RH, McGrath JM, Reyna MBA, McCain N, Lewis MM, Cone MS (2010). A model of neurodevelopmental risk and protection for preterm infants.

[10] Als H, Duffy FH, McAnulty GB, Rivkin MJ, Vajapeyam S, Mulkern RV (2004). Early experience alters brain function and structure. Pediatrics..

[11] Peters KL, Rosychuk RJ, Hendson L, Cote JJ, McPherson C, Tyebkhan JM (2009). Improvement of short- and long-term outcomes for very low birth weight infants: Edmonton NIDCAP trial. Pediatrics..

[12] Anand K, Scalzo FMJN (2000). Can adverse neonatal experiences alter brain development and subsequent behavior?.

[13] Barnard KE, Brazelton TB (1990). Johnson Pediatric Round T. Touch : the foundation of experience.

[14] Gorski PA, Leonard C H, Sweet D M, Martin J A (1990). Caregiver-infant interaction and the immature nervous system: a touchy subject.

[15] Berger SP, Holt-Turner I, Cupoli JM, Mass M (1998). Hageman JRJPCoNA Caring for the graduate from the neonatal intensive care unit: at home, in the office, and in the community.

[16] Brett J, Staniszewska S, Newburn M, Jones N, Taylor LJBo (2011). A systematic mapping review of effective interventions for communicating with, supporting and providing information to parents of preterm infants.

[17] Orton J, Spittle A, Doyle L, Anderson P, Boyd RJDM, Neurology C (2009). Do early intervention programmes improve cognitive and motor outcomes for preterm infants after discharge? A systematic review.

[18] Spittle A, Orton J, Anderson PJ, Boyd R, Doyle LWJCDoSR (2015). Early developmental intervention programmes provided post hospital discharge to prevent motor and cognitive impairment in preterm infants.

[19] Symington A, Pinelli JJTCdosr (2003). Developmental care for promoting development and preventing morbidity in preterm infants.

[20] Symington A, Pinelli JJTCdosr Developmental care for promoting development and preventing morbidity in preterm infants.

[21] Aita M, Stremler R, Feeley N, Lavallée A, De Clifford-Faugère GJSr (2017). Effectiveness of interventions during NICU hospitalization on the neurodevelopment of preterm infants: a systematic review protocol.

[22] Lavallée A, De Clifford-Faugère G, Garcia C, Oviedo ANF, Héon M, Aita MJJoNN (2018). Part 1: Narrative overview of developmental care interventions for the preterm newborn.

[23] Jacobs SE, Sokol J, Ohlsson A (2002). The newborn individualized developmental care and assessment program is not supported by meta-analyses of the data. The Journal of Pediatrics..

[24] Moher D, Shamseer L, Clarke M, Ghersi D, Liberati A, Petticrew M (2015). Preferred reporting items for systematic review and meta-analysis protocols (PRISMA-P) 2015 statement.

[25] Bayley N (1969). Manual for the Bayley scales of infant development.

[26] Bayley N (1993). Bayley scales of infant development: Manual.

[27] Bayley N ( 2006). Bayley scales of infant and toddler development: Bayley-III.

[28] Higgins JPT, Altman DG, Gøtzsche PC, Jüni P, Moher D, Oxman AD (2011). The Cochrane Collaboration’s tool for assessing risk of bias in randomised trials.

[29] Rucker G, Cates CJ, Schwarzer G (2017). Methods for including information from multi-arm trials in pairwise meta-analysis. Research synthesis methods..

[30] Ryan RE, Hill SJ, Prictor M, J M (2013). Cochrane Consumers and Communication group Study Quality Guide.

